# Heterogeneous Cellular Response of Primary and Metastatic Human Gastric Adenocarcinoma Cell Lines to Magnoflorine and Its Additive Interaction with Docetaxel

**DOI:** 10.3390/ijms242115511

**Published:** 2023-10-24

**Authors:** Aneta Grabarska, Jarogniew J. Luszczki, Kinga Gawel, Wirginia Kukula-Koch, Małgorzata Juszczak, Adrianna Slawinska-Brych, Grzegorz Adamczuk, Magdalena Dmoszynska-Graniczka, Nataliia Kosheva, Wojciech Rzeski, Andrzej Stepulak

**Affiliations:** 1 Department of Biochemistry and Molecular Biology, Medical University of Lublin, Chodzki 1, 20-093 Lublin, Poland; magdalena.dmoszynska-graniczka@umlub.pl; 2 Department of Occupational Medicine, Medical University of Lublin, Jaczewskiego 8b, 20-090 Lublin, Poland; jarogniew.luszczki@umlub.pl; 3 Department of Experimental and Clinical Pharmacology, Medical University of Lublin, Jaczewskiego 8b, 20-090 Lublin, Poland; kingagawel@umlub.pl (K.G.); nataliia.kosheva21@gmail.com (N.K.); 4 Department of Pharmacognosy with Medicinal Plants Garden, Medical University of Lublin, Chodzki 1, 20-093 Lublin, Poland; virginia.kukula@gmail.com; 5 Department of Medical Biology, Institute of Rural Health, Jaczewskiego 2, 20-090 Lublin, Poland; juszczak.malgorzata@imw.lublin.pl (M.J.); rzeski.wojciech@imw.lublin.pl (W.R.); 6 Department of Cell Biology, Institute of Biological Sciences, Maria Curie-Skłodowska University, Akademicka 19, 20-033 Lublin, Poland; adrianna.slawinska-brych@mail.umcs.pl; 7 Independent Medical Biology Unit, Medical University of Lublin, Jaczewskiego 8b, 20-090 Lublin, Poland; grzegorz.adamczuk@umlub.pl; 8 Department of Functional Anatomy and Cytobiology, Maria Curie-Skłodowska University, Akademicka 19, 20-033 Lublin, Poland

**Keywords:** gastric cancer, magnoflorine, *Berberis vulgaris*, proliferation, cell cycle, apoptosis, drug interactions, zebrafish

## Abstract

Gastric cancer is the most common cancer and remains the leading cause of cancer death worldwide. In this study, the anticancer action of magnoflorine isolated via counter-current chromatography from the methanolic extract of *Berberis vulgaris* root against gastric cancer in models of primary ACC-201 and AGS and metastatic MKN-74 and NCI-N87 cell lines was analyzed. Cell viability and proliferation were tested through the use of MTT and BrdU tests, respectively. Cell cycle progression and apoptosis were evaluated using flow cytometry. The interaction of magnoflorine and docetaxel has been examined through isobolographic analysis. Moreover, potential toxicity was verified in zebrafish in an in vivo model. Gastric cancer cell lines revealed different responses to magnoflorine treatment with regard to viability/proliferation, apoptosis induction and cell cycle inhibition without any undesirable changes in the development of larval zebrafish at the tested concentrations. What is more, magnoflorine in combination with docetaxel produced an additive pharmacological interaction in all studied gastric cancer cell lines, which may suggest a complementary mechanism of action of both compounds. Taken together, these findings provide a foundation for the possibility of magnoflorine as a potential therapeutic approach for gastric cancer and merits further investigation, which may pave the way for clinical uses of magnoflorine.

## 1. Introduction

Based on GLOBOCAN 2020 data, gastric cancer (GC) is the 5th most common cancer and remains the leading cause of cancer death worldwide [1]. The incidence and mortality rates for GC vary between countries and regions [2] and are often related to environmental factors such as dietary components, lifestyle and *Helicobacter pylori* infection [3]. The poor relative survival rate of patients with GC usually reflects the fact that most cases of GC are identified at the metastatic or unresectable stage [4]. The commonly used therapies for advanced GC are radiotherapy, chemotherapy and targeted therapy based on agents such as imatinib, larotrectinib, entrectinib and regorafenib [5]. However, despite the dynamic progress, oncological therapy still brings with it some inevitable consequences, such as a low response rate, the disseminated resistance of cancer cells to chemotherapeutic agents and many adverse drug reactions [6]. Therefore, there is a need for further efforts toward finding alternative and efficient therapies to improve the survival and quality of life of GC patients.

Herbal plants are the oldest source of pharmacotherapy compounds [7], and they are still being used to treat diseases or as alternatives to many synthetic drugs used in clinical therapies [8]. It has been well documented that plants, due to their rich source of structurally diverse bioactive compounds, such as alkaloids, flavonoids, lignans, saponins, terpenes, taxanes, glycosides, gums and oils [9], may offer an opportunity to discover new agents with a broad spectrum of therapeutic activities. Currently, multiple plants and plant-derived substances that have already been proven to be effective against one or more types of cancers have been described. Among well-known compounds isolated from plants and used in cancer treatment are paclitaxel, vincristine and vinblastine [10]. 

Common barberry (*Berberis vulgaris* L.) is a shrub of particular interest due to its proven nutritional value and medicinal benefits [11]. Phytochemical analysis of *Berberis vulgaris* roots revealed the presence of a secondary metabolite, namely magnoflorine (MGN), in its extracts [12]. MGN, also named thalictrine and escholine, is a quaternary benzylisoquinoline alkaloid that is characterized by the presence of an aporphine scaffolding, and it is found in numerous plant species belonging to the Berberidaceae, Magnoliaceae, Menispermaceae, Papaveraceae, or Ranunculaceae families [13]. MGN is increasingly attracting research attention because of its wide spectrum of pharmacological activities, including anti-diabetic, anti-inflammatory, neuropsychopharmacological, immunomodulatory, hypotensive, antioxidant and antifungal activities [14]. It has also been shown that MGN suppresses the growth of a few types of cancer, including breast cancer [15,16], cervical cancer, brain tumors, hepatocellular carcinoma [17], non-small cell lung cancer, colorectal cancer, pancreatic cancer [18], GC [19] and osteosarcoma [20]. 

MGN was isolated through the use of counter-current chromatography from the methanolic extract of *Berberis vulgaris*, which is commonly grown in Europe. To the best of our knowledge, only one report by Sun et al. [19] indicates that MGN reduces the cell proliferation of GC cell lines. It has been shown that MGN induced autophagy, apoptosis and cell cycle arrest through the activation of the c-Jun N-terminal kinase (JNK) signaling pathway regulated by reactive oxygen species in human GC SGC-7901 cells, the most sensitive of the tested cell lines [19]. We investigated the biological activities of MGN against adenocarcinoma GC cell lines that were established from both primary tumors (well-differentiated ACC-201 [21] and moderately differentiated AGS [22,23]) and metastatic sites (moderately differentiated MKN-74 [24] and well-differentiated NCI-N87 [22,24,25]). A series of experiments were carried out to examine the differences in the action of MGN between the studied GC cell lines. Furthermore, the interaction of MGN with docetaxel (DCT), a cytostatic routinely used in GC therapy, was tested through the use of isobolographic analysis. The effect of MGN on morphology, touch response (muscle function and performance) and the basic locomotor activity of larval zebrafish were analyzed.

## 2. Results

### 2.1. MGN and DCT Impact on the Viability/Proliferation of ACC-201, AGS, MKN-74 and NCI-N87 Human GC Cell Lines in a Dose-Dependent Manner

**Figure 1:**
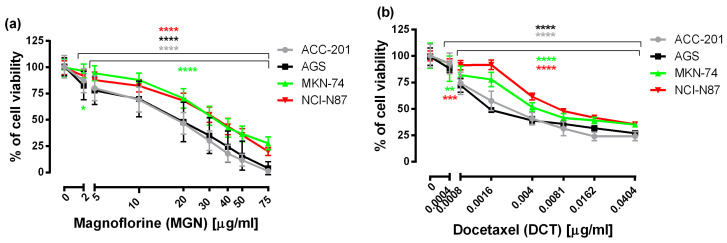
The concentration-related inhibitory effects of MGN (**a**) and DCT (**b**) on the viability of human GC cells incubated with selected concentrations of these compounds for 72 h. Results are expressed as the mean ± the standard error of the mean (SEM) of 24 samples (n = 24) from at least three independent experiments. Statistical differences were analyzed with Student’s *t*-test (* *p* < 0.05; ** *p* < 0.01; *** *p* < 0.001; **** *p* < 0.0001 vs. control).

First, to examine the effectiveness of MGN and DCT toward different human GC cell lines, including ACC-201, AGS, MKN-74 and NCI-N87, the 3-(4,5-dimethylthiazol-2-yl)-2,5-diphenyltetrazolium bromide (MTT) assay was used, which measures the mitochondrial succinate dehydrogenase activity of cells. As shown in Figure 1, the viability of GC cells was significantly reduced in the whole range of MGN and DCT concentrations tested, and the observed effect was dose-dependent. 

From the linear log–probit concentration–response inhibitory effects for MGN and DCT on four cell lines, it was possible to determine the median inhibitory concentrations (IC_50_ values ± SEM). These data showed that GC ACC-201 and AGS cells were, on average, more sensitive to MGN than MKN-74 and NCI-N87 cells derived from GC metastases to the liver. The IC_50_ values of MGN for the human GC cell lines ACC-201, AGS, MKN-74 and NCI-N87 were 15.75 µg/mL, 17.19 µg/mL, 34.82 µg/mL and 33.31 µg/mL, respectively. A similar tendency has been observed in the action of DCT. The IC_50_ values of DCT for the human GC cell lines ACC-201, AGS, MKN-74 and NCI-N87 were 0.0028 µg/mL, 0.0026 µg/mL, 0.0049 µg/mL and 0.0090 µg/mL, respectively (Table 1).

**Figure 2:**
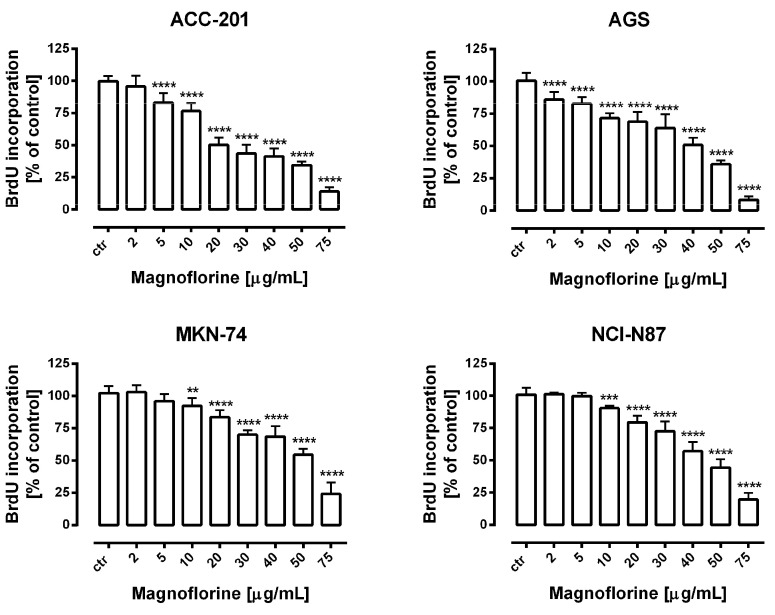
The concentration-dependent inhibitory effects of MGN on the proliferation of human GC cells incubated with selected concentrations of this compound (2–75 μg/mL) for 72 h. Results are expressed as the mean ± SEM of 24 samples from at least three independent experiments. Statistical differences were analyzed using Student’s *t*-test (** *p* < 0.01; *** *p* < 0.001; **** *p* < 0.0001 vs. control).

Further, the anti-proliferative properties of MGN were verified using a more sensitive and specific method based on the measurement of DNA synthesis. In our studies, we used bromodeoxyuridine (BrdU), a thymidine analogue, which is incorporated into the S-phase cells of newly synthesized DNA [26]. A dose-dependent decrease in BrdU-labeled DNA was noted in the MGN-treated GC cell lines. Importantly, the obtained data were statistically significant and corresponded with the results of MTT studies in concentration ranges of MGN from 10 to 75 µg/mL (Figure 2). Therefore, we considered the application of these doses of MGN to investigate its mechanism of action against GC cell lines.

### 2.2. MGN Treatment Induces Apoptosis in ACC-201, AGS, MKN-74 and NCI-N87 Human GC Cell Lines

**Figure 3:**
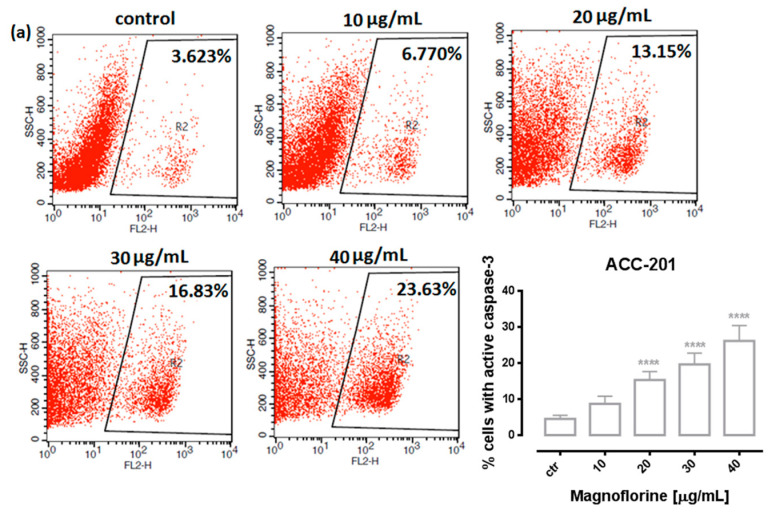

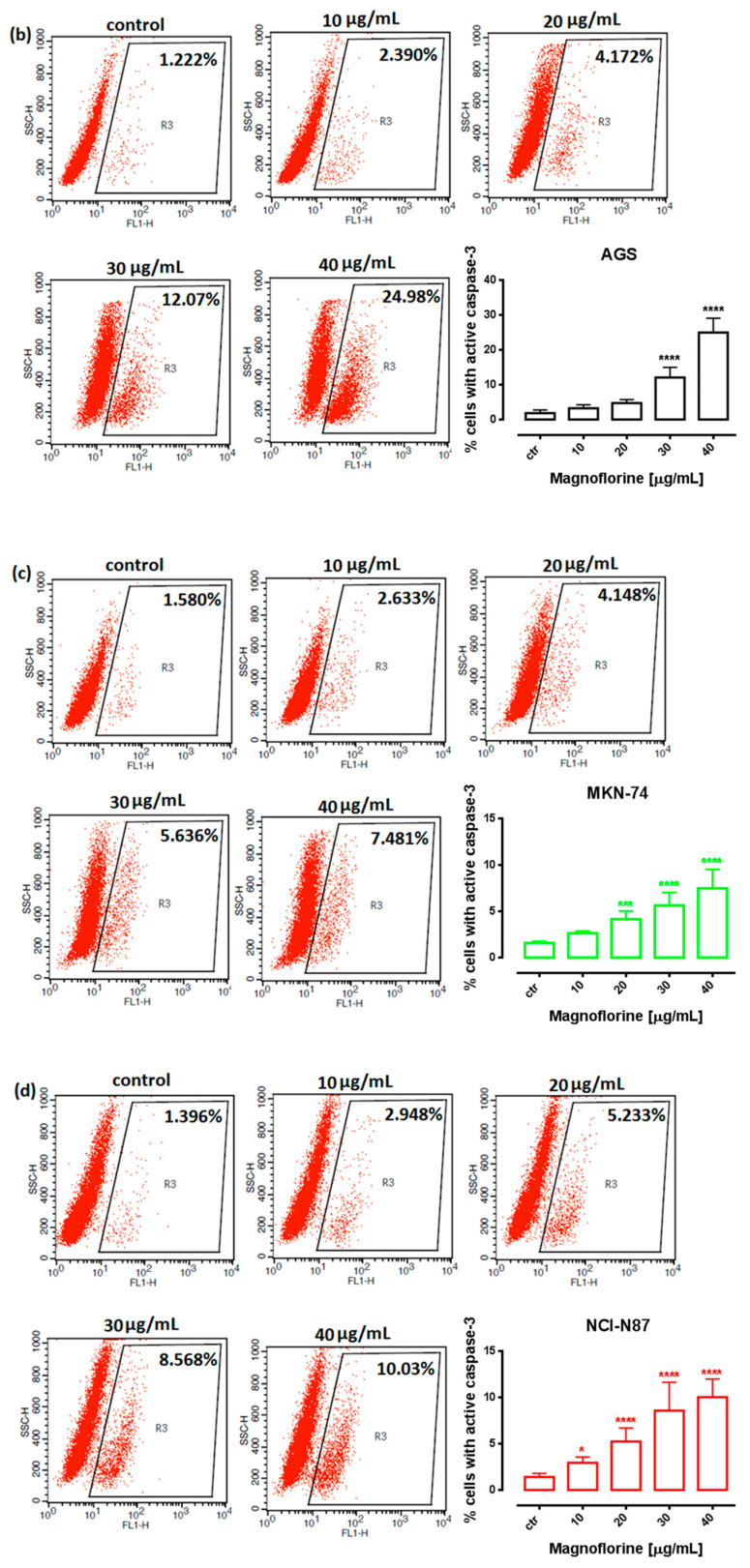
Representative flow cytometry dot plot graphs of ACC-201 (**a**), AGS (**b**), MKN-74 (**c**) and NCI-N87 (**d**) GC cell lines after the treatment with a medium (ctr) and MGN for 72 h. Regions R2 and R3 included apoptotic cells with active caspase-3. All results are expressed as mean ± SEM of three independent experiments. Statistical differences were analyzed using Student’s *t*-test (* *p* < 0.05; *** *p* < 0.001; **** *p* < 0.0001 vs. control).

In order to investigate the possible route of cell death caused by MGN, we analyzed the activity of caspase-3. In a dose-dependent manner, MGN treatment resulted in an increase in the number of cleaved caspase-3-positive cells in all of the studied GC cell lines compared with the control, that is, untreated GC cells (Figure 3a–d).

**Figure 4:**
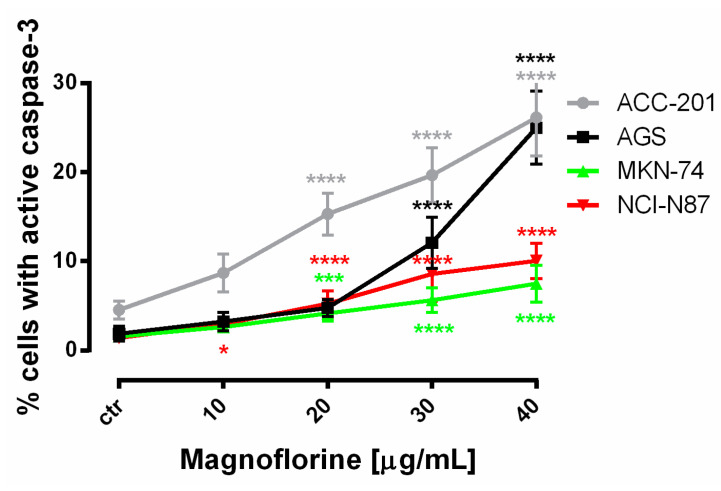
The number of cleaved caspase-3-positive ACC-201, AGS, MKN-74 and NCI-N87 GC cell lines after the treatment with a medium (ctr) and MGN for 72 h. All results are expressed as mean ± SEM of three independent experiments. Statistical differences were analyzed using Student’s *t*-test (* *p* < 0.05; *** *p* < 0.001; **** *p* < 0.0001 vs. control).

Similar to MTT and BrdU assays, FACS analysis showed that ACC-201 and AGS cell lines were more sensitive and showed more apoptotic cells after treatment with MGN than metastatic MKN-74 and NCI-N87 cell lines (Figure 4 and Table 2).

### 2.3. MGN Treatment Induces Changes in the Cell Cycle Progression of ACC-201, AGS, MKN-74 and NCI-N87 Human GC Cell Lines

**Figure 5:**
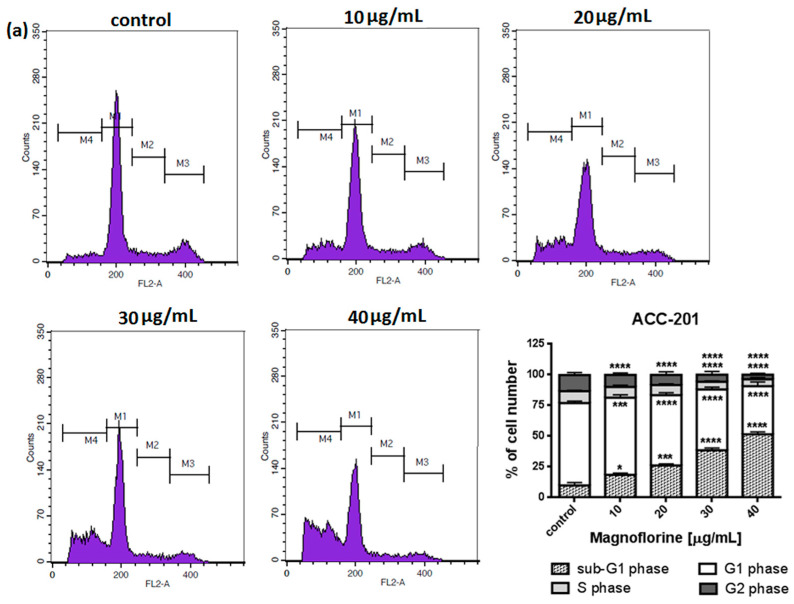

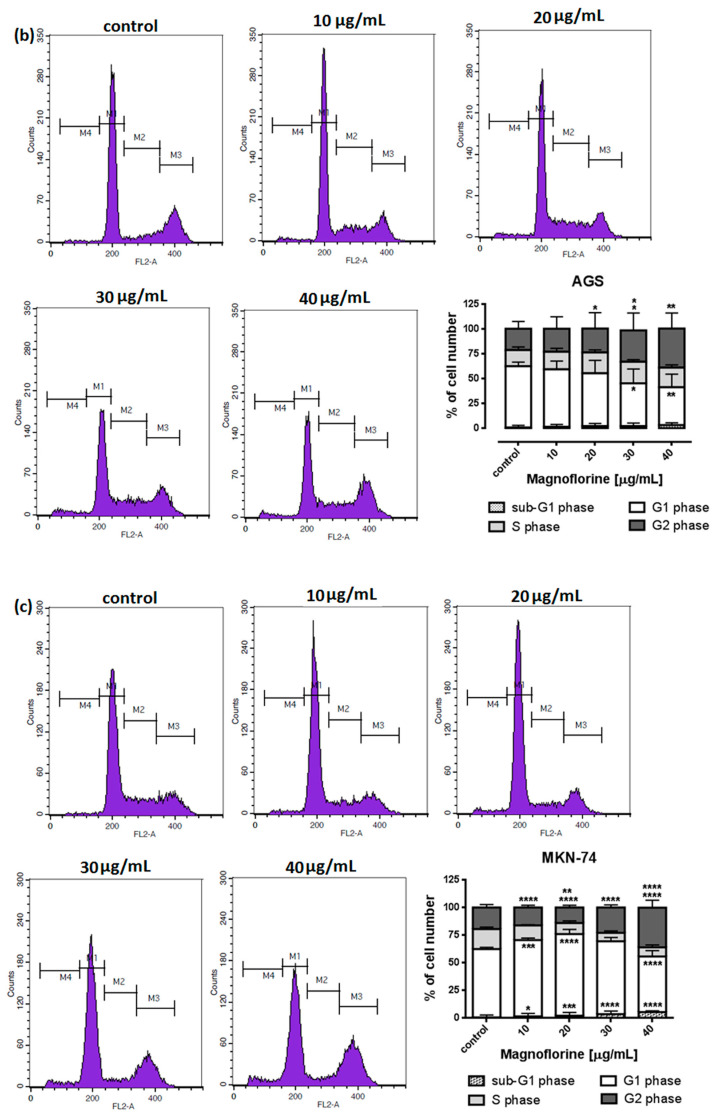

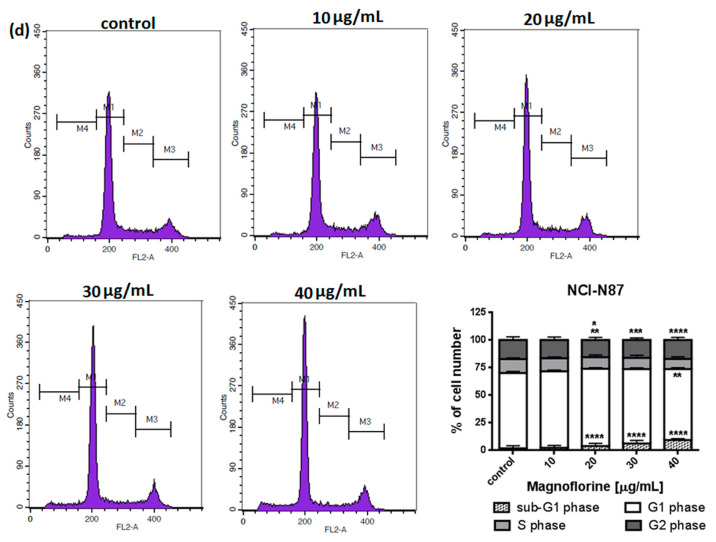
Representative cell cycle progression of the human GC cell lines following MGN treatments for 72 h using a flow cytometer. Representative histograms of PI-stained nuclear DNA content of each cell cycle phase vs. cell counts in untreated control and treated ACC-201 (**a**), AGS (**b**), MKN-74 (**c**) and NCI-N87 (**d**) cells, respectively. M1, M2, M3 and M4 in each histogram denote the number of cells in sub-G1, G1, S and G2/M phase, respectively. All results were expressed as means ± SEM of three independent experiments. Statistical differences were analyzed using Student’s *t*-test (* *p* < 0.05; ** *p* < 0.01; *** *p* < 0.001; **** *p* < 0.0001 vs. control).

To explore the functional mechanism of MGN in inhibiting cell growth, we also analyzed whether MGN leads to alterations in the cell cycle. Thus, the cells were treated with selected concentrations of MGN and stained with propidium iodide (PI). The percentages of cells in each stage of the cell cycle (sub-G1, G0/G1, S and G2/M) were determined through the use of flow cytometry. All analyzed cell lines responded differently to MGN treatment. The mean proportions of the subG1-phase were clearly higher in the ACC-201 cell line than in other MGN-treated GC cells (Figure 5a and Table 3). MGN induced the G2/M phase arrest of AGS cells in a dose-dependent manner (Figure 5b and Table 3). Depending on the dose of MGN applied (10 and 20 µg/mL vs. 40 µg/mL), the treatment of metastatic MKN-74 cells induced G1/S or G2/M phase cell cycle arrest compared to non-treated MKN-74 cells. (Figure 5c and Table 3). Minor changes in cell cycle progression were observed in the other metastatic NCI-N87 cell line, restricted to the slight increase in the number of cells in the sub-G1 phase of the cell cycle (Figure 5d and Table 3).

### 2.4. Anti-Proliferative Effect of DCT and MGN Administered in Combination in GC Cell Lines (ACC-201, AGS, MKN-74 and NCI-N87)

**Figure 6:**
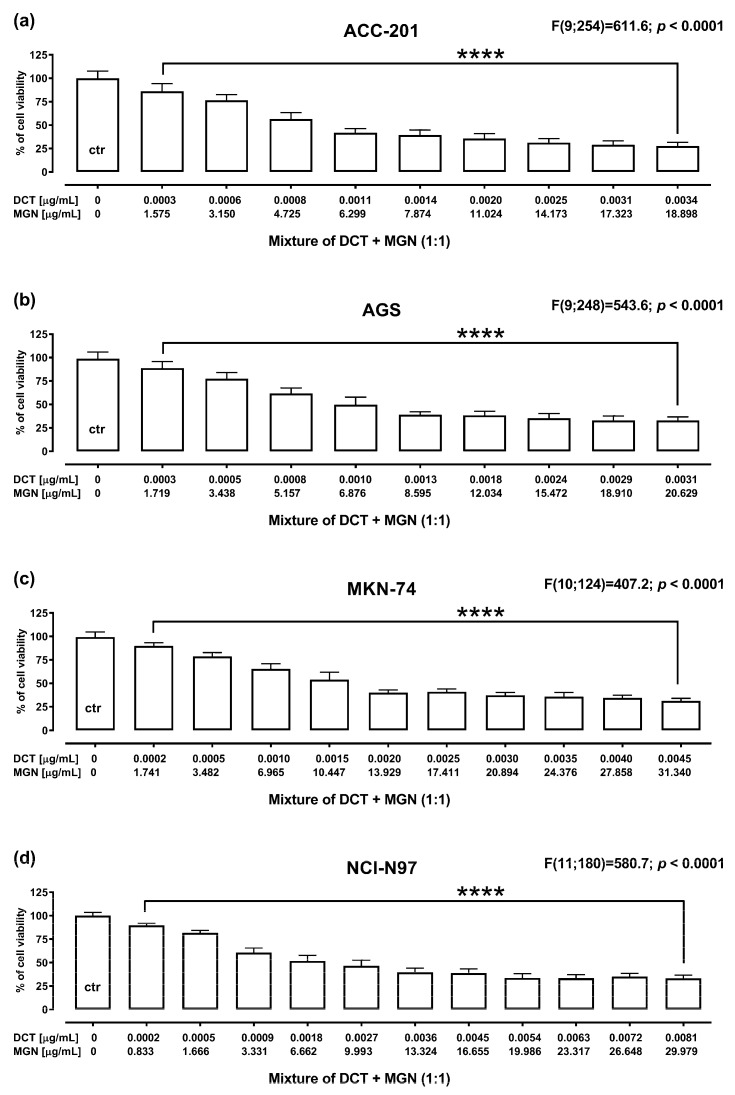
The anti-proliferative effect for the mixture of DCT and MGN administered in combination in ACC-201 (**a**), AGS (**b**), MKN-74 (**c**) and NCI-N87 (**d**) cell lines. Cell viability for the mixture of DCT and MGN administered for 72 h at the fixed ratio of 1:1 was measured by the MTT assay with various increasing concentrations of both active agents. All GC cell lines were exposed to DCT and MGN mixture treatment using different ratios of their IC_50_ values. Data are expressed as mean ± SEM. All results were analyzed using a one-way ANOVA test followed by Dunnett’s multiple comparisons test. Statistical differences were considered relevant at **** *p* < 0.0001 vs. the control group.

The anti-proliferative effect of DCT and MGN administered in combination for 72 h against GC cell lines was assessed using MTT assay. DCT and MGN were administered in combination with a 1:1 drug mixture in increasing concentrations. All GC cell lines were exposed to the DCT and MGN mixture treatment. The results are presented as the means (in μg/mL) ± SEM of DCT and MGN administered in combination with respect to their anti-proliferative effects in four GC cell lines (ACC-201, AGS, MKN-74 and NCI-N87) (Figure 6).

### 2.5. Analysis of Pharmacological Interaction between MGN and DCT Using Isobolographic Method

**Figure 7:**
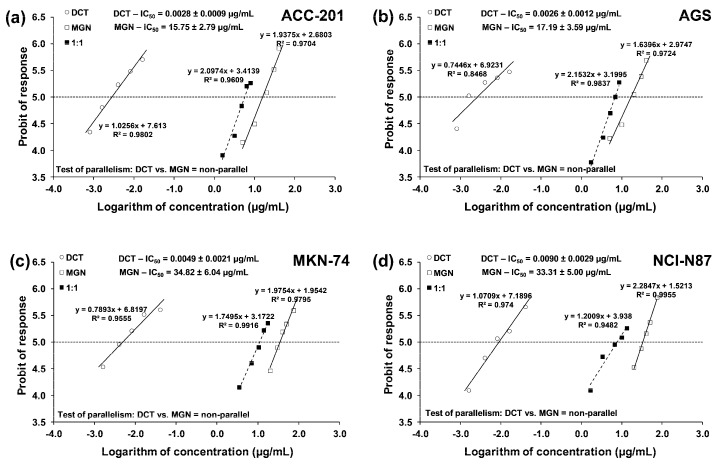
Concentration–effect lines for MGN and DCT administered alone and in combination for 72 h at the fixed-ratio of 1:1, illustrating the anti-proliferative effects of the drugs and their mixture in ACC-201 (**a**), AGS (**b**), MKN-74 (**c**) and NCI-N87 (**d**) cell lines, measured in vitro through the use of the MTT assay. The dashed line on each graph represents in approx. the IC_50_ values of the studied drugs administered either alone or in combination.

The test for parallelism confirmed that the experimentally determined concentration–effect lines for MGN and DCT (administered alone) are mutually non-parallel to each other in four various GC cell lines (Figure 7a–d).

**Figure 8:**
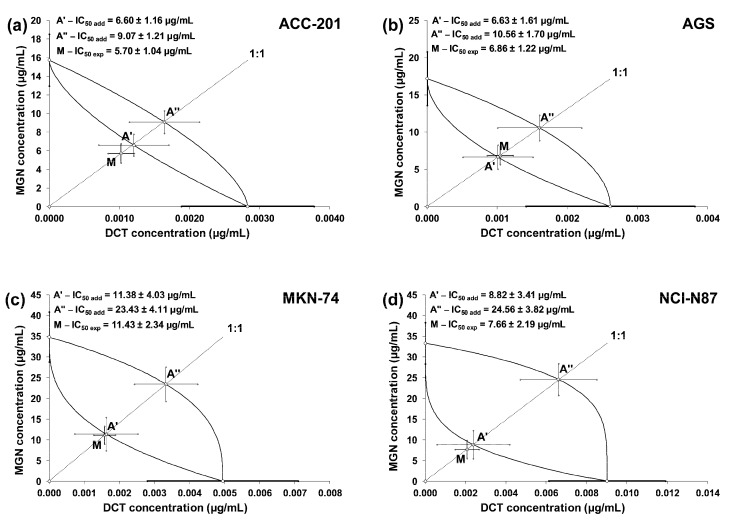
Isobolograms illustrating additive interactions between MGN and DCT with respect to their anti-proliferative effects on ACC-201 (**a**), AGS (**b**), MKN-74 (**c**) and NCI-N87 (**d**) cell lines measured in vitro using the MTT assay for 72 h. The IC_50_ ± SEM for MGN and DCT are plotted on the Y- and X-axes, respectively. The points A′ and A″ depict the theoretically calculated IC_50add_ values (±SEM) for both lower and upper isoboles of additivity. The point M on each graph represents the experimentally derived IC_50mix_ value (±SEM) for the mixture, which produced a 50% anti-proliferative effect (50% isobole) in the tested cell lines.

The linearly related concentration–response effects for the studied drugs (when used alone and in combinations) were plotted into the Cartesian system of coordinates to create isobolograms. The combinations of MGN and DCT at a fixed ratio of 1:1 showed additive interactions for every tested cell line. Unfortunately, no synergistic interaction was confirmed via a statistical comparison of the IC_50mix_ value (point M on the isobologram) with the corresponding IC_50add_ value (point A′) with Student’s *t*-test with Welch’s correction (Table 4). On the isobolograms, the proximity of point M to point A′ indicates an additive interaction (Figure 8a–d).

### 2.6. Developmental Toxicity of MGN in Zebrafish

Zebrafish embryos and larvae were incubated from 1 up to 96 h post-fertilization (hpf) in different MGN concentrations. Table 5 shows the effect of MGN on the hatching rate (%) of larvae after 71 or 95 h of incubation.

Here, larvae exposed to 100 µg/mL or 175 µg/mL MGN concentration did hatch within the same time frame as control counterparts (100% for both concentrations), i.e., within 72 h. For the concentration of 250 µg/mL MGN, we observed the delay in the hatching rate at 72 hpf (52.77%), but the majority of larvae hatched up to 96 hpf (94.44%). In this study, 86.66% of larvae exposed to 500 µg/mL MGN did hatch after 96 h, but only 23.33% hatched after 72 h, which means there were significantly delayed in terms of development compared to the control larvae. In a concentration of 750 µg/mL MGN, none of the larvae hatched up to 96 h, although all survived in chorions.

**Figure 9:**
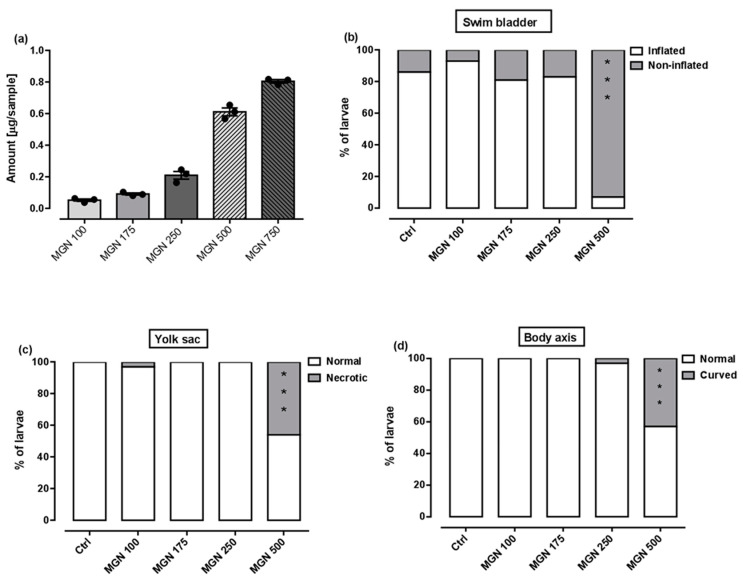

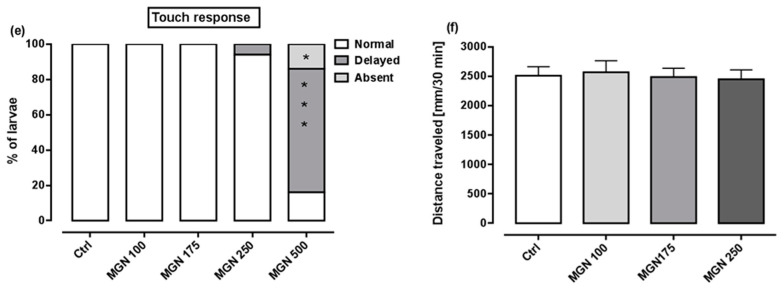
(**a**) Amount of MGN (concentrations: 100, 175, 250, 500 or 750 µg/mL) absorbed by zebrafish larvae after 95 h of exposure (n = 3 samples per group, n = 50 larvae per sample). Effect of MGN (concentrations: 100, 175, 250 or 500 µg/mL) on: (**b**) swim bladder inflation, (**c**) yolk sac necrosis, (**d**) body axis and (**e**) touch response at 96 hpf (n = 27–31 per group) (* *p* < 0.05, *** *p* < 0.001 vs. control group; Fisher’s exact test). (**f**) Effect of MGN (concentrations: 100, 175 or 250 µg/mL) on locomotor activity of larvae after 95 h long incubation (one-way ANOVA). Ctrl: control; MGN: magnoflorine.

**Figure 10:**
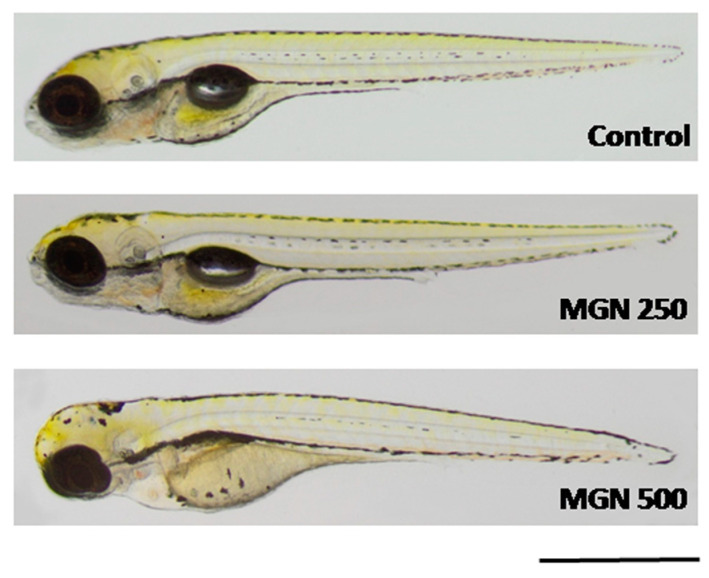
Representative photographs of larvae incubated in MGN (250 or 500 µg/mL). MGN:magnoflorine. Scale bar: 1 mm.

Figure 9a shows that in the range of tested concentrations, MGN was absorbed by the larvae after 95 h long incubation (for representative photos of larvae, see Figure 10). Since none of the larvae hatched at a concentration of 750 µg/mL MGN after 95 h of incubation, we did not study this group in subsequent experiments. Through morphological analysis, we clearly showed that the three lowest concentrations did not affect any of the parameters scored. Larvae exposed chronically to 500 µg/mL MGN were substantially underdeveloped. We observed the lack of swim bladder in 93% (*p* < 0.001), yolk sac necrosis in 46% (*p* < 0.001) and curved body axis in 43% (*p* < 0.001) of MGN-treated larvae, compared to the control counterparts. Additionally, we have seen heart edema in 40% (*p* < 0.001) of the MGN-exposed larvae and underdeveloped jaw in the majority of them (90%; *p* < 0.001). Additionally, 70% of the larvae incubated in 500 µg/mL MGN (*p* < 0.001) had delayed touch response, which means their muscle performance was disturbed. Therefore, we did not investigate the effect of that concentration through the use of a locomotor activity assay, as this could have yielded false-positive results.

A locomotor activity assay was performed after chronic exposure to MGN (100, 175 or 250 µg/mL) to determine whether MGN may exert neurotoxic effects. No differences between the tested groups in regard to the distance travelled within the 30 min observation period were seen (one-way ANOVA: F(3,92) = 0.09, *p* > 0.05; Figure 9f).

## 3. Discussion

GC remains one of the most aggressive, hard-to-treat types of cancer, and is often characterized by late diagnosis and incredibly poor survival rates [27]. There is an increased interest in searching for new effective anti-cancer drugs sourced from natural compounds. It has been shown that MGN possesses anti-tumor activities in multiple cancers, both in vitro and in vivo, which are associated with its effects on multiple signaling pathways that promote cell cycle arrest [16,19], the induction of apoptosis [16,19] and autophagy [19], epithelial–mesenchymal transition [20], invasion and metastasis [20]. Studies conducted by Wang et al. [20] and Wei et al. [28] have shown that MGN significantly reduced the viability/proliferation of MG-63 and U-2 osteosarcoma cell lines and MCF-7, MDA-MB-231, MDA-MB-453 and BT474 breast cancer cell lines, respectively. It was found that MGN, without harmful pharmacokinetic effects [18], may be a potent inhibitor of the epidermal growth factor receptor (EGFR), which is one of oncology’s primary targets and is crucial for cell growth, proliferation, survival and differentiation [18,29]. Our study revealed that MGN remarkably suppressed the growth of GC cell lines in a dose-dependent manner. We found that the human GC cell lines MKN-74 and NCI-N87 derived from liver metastasis of gastric carcinoma were more resistant to MGN. However, the level of growth inhibition was not related to the primary differentiation degree of each cancer cell line. The observed significant differences in responses to MGN between analyzed GC cell lines prove that metastatic cancer is notoriously difficult to treat, and that it accounts for most cancer deaths [30]. The treatment of metastases urgently needs improvement as patients with distant metastases of GC have a five-year survival of only 3.9% compared to 70% for patients with local disease [31]. A substantial decrease in growth and metabolic activity in response to MGN has also been demonstrated in other types of human GC cell lines, such as MGC-803, BGC-823 and SGC-7901 [19]. The inhibition of cell viability was most notable in the SGC-7901 cancer cell line established from the metastatic lymph node of a patient with stage 4 GC with remarkable peritoneal invasion [32]. GC cell lines BGC-823 and SGC-7901 have been widely used in GC-related studies. However, it should be noted that both cell lines have been described as problematic and contaminated or have been shown to be HeLa derivatives [33]. Therefore, results based on them might be questionable.

The deregulation of apoptotic pathways and cell cycle are common features of cancer cells [34]. In our studies, the potential of MGN to suppress cell division and induce cell cycle perturbation was initially confirmed via the dose-dependent inhibition of DNA synthesis. Flow cytometry analysis indicated that the changes in cell cycle progression seem to be dependent on cell type and MGN concentration. MGN displayed anti-proliferative action against AGS cells by blocking the cell cycle at the G2/M phase. A dual response was shown in the metastatic MKN-74 cell line. The transition of MKN-74 cells from G1 to the S phase was delayed or inhibited, followed by an increase in the number of cells in the G2/M phase parallel to the increasing concentrations of MGN. MGN-mediated mitotic arrest was also reported in SGC-7901 GC [19] and NCI-H1299 lung cancer, MDA-MB-468 breast cancer and T98G glioma cancer cells [16]. Moreover, it has been found that the mechanism by which MGN contributes to cell-cycle arrest in the G2/M phase and, thus, growth suppression was mediated by the up-regulation of the CDK’s kinases inhibitor proteins p21^Waf1/Cip1^ and p27^Kip1^ and the down-regulation of Cyclin-A and Cyclin-B1 [19]. The dose-dependent increase in the sub-G1 phase in ACC-201 and NCI-N87 GC cells after treatment with MGN may prove the ability of MGN to trigger apoptotic cell death. Indeed, we have shown that MGN triggered apoptosis in all studied GC cell lines in a dose-dependent manner, especially in ACC-201 and AGS primary GC cell lines. This is in agreement with the MTT assay data showing differences in the sensitivity of GC cells to MGN. Heterogeneous responses of GC cell lines to MGN treatment with regard to viability/proliferation and cell cycle inhibition, as well as apoptosis induction, might be attributed to the specific genomic mutations harbored by the cells. The altered *TP53* gene, which encodes for the p53 tumor suppressor protein, has been recognized as one of the most frequent mutations in GC [35]. Moreover, p53 mutation status has been shown to affect the cellular response induced by cytotoxic drugs [36]. We have found that cell lines with mutated p53, including MKN-74 (I251L) [37,38] and NCI-N87 (R248Q) [39,40], were less sensitive to MGN compared to AGS cells that express the wild type of p53 [41]. Anyway, further studies are needed to examine that issue.

Accumulating evidence indicates that the administration of natural products affecting multiple biochemical and molecular targets [42,43] with chemotherapeutic drugs maximized their efficacy while minimizing potential side effects [44,45]. In keeping with the authors above, we have examined the effect of MGN in enhancing the efficacy of DCT treatment in GC cell lines. DCT-based triplet chemotherapies are one of the commonly used regimens in progressive GC. However, despite their efficacy, it has been reported that DCT regimens are not well-tolerated due to their high rate of toxicity [46,47,48]. Using isobolographic analysis, a statistically robust method for the quantitative assessment of drug combinations, we have found that MGN and DCT used together at a fixed ratio of 1:1 produced an additive pharmacological interaction in all of the studied GC cell lines, which may suggest a complementary mechanism of action of both compounds. The enhanced effect of DCT due to MGN observed in studied GC cell lines allows us to believe that the combinatorial treatment could lead to better benefits than those observed during the unique treatment with DCT.

The beneficial effects of the concomitant administration of MGN with the other cytostatic drugs, such as cisplatin (CDDP) and doxorubicin (DOX), have been observed against various tumor types. The mixture of CDDP and MGN exerted additive or supra-additive (synergistic) interactions in TE671 human rhabdomyosarcoma, T98G human glioblastoma, MDA-MB-468 human triple-negative breast cancer and NCI-H1299 non-small lung cancer cell lines [49]. MGN also enhanced the CDDP sensitivity of MG-63 and U-2 osteosarcoma cells via the suppression of HMGB1/NF-κB signaling [20]. Moreover, MGN/DOX co-treatment dose-dependently decreased breast cancer growth, both in vitro and in vivo, without cytotoxicity to the MCF-10A normal breast cancer cell line and significant side effects to the animals. Notably, combining MGN with DOX exerted a synergistic anti-tumor mode of action in MCF-7 and MDA-MB-231 breast cancer cell lines. It has also been shown that MGN/DOX combinational treatment significantly induced apoptosis through a mitochondria-dependent pathway and resulted in the induction of autophagy through the up-regulation of beclin-1 and light chain 3 (LC3)-II expression regulated by the phosphoinositide 3-kinase/Akt and p38 mitogen-activated protein kinase signaling pathways [28]. Unfortunately, in this study, we observed no synergistic collaboration of MGN with DCT in inhibiting the growth of primary and metastatic GC cell lines. In such a situation, we are forced to accept the additive nature of the interactions between MGN and DCT in the various studied GC (ACC-201, AGS, MKN-74 and NCI-N87) cell lines.

Importantly, potential therapeutic agents often fail in vivo testing since they have inherent toxicity and poor pharmacokinetic properties that are undetectable in in vitro studies [50]. The most common and attractive biological model to determine the safety of natural products in vivo is zebrafish. Embryos and larvae are preferred due to their small size and optical transparency, which allows the direct observation of the developmental stages and noninvasive visualization of the internal organs [51]. In this study, we assessed the effect of chronic exposure to MGN on the development of larval zebrafish, starting at 1 hpf. To the best of our knowledge, this is the first study aimed at determining the safety of MGN during developmental processes. We observed that MGN was safe for developing zebrafish in a rather narrow range of concentrations—a concentration of 100 µg/mL seemed to be safe, but a five-times higher dose caused different abnormalities. We did not evaluate the locomotor activity of those larvae, but fish exposed to 250 µg/mL MGN and lower performed similarly to control counterparts in terms of a locomotor activity assay; that is, in that range of concentrations, MGN did not exert neurotoxic effects. Thereby, we may conclude that up to 250 µg/mL MGN, no evidence of toxicity exists, but caution is needed.

## 4. Materials and Methods

### 4.1. Compounds

MGN with a purity of 96.2% was isolated in the previously published protocol [12] from the dried ground root of *Berberis vulgaris* produced by Proherbis company (Debowiec, Poland), which was purchased in Lublin (Poland) in February 2023 in a herbal shop. The methanolic extract from the plant material was prepared as described before using the accelerated solvent extractor (ASE100, Dionex, Sunnyvale, CA, USA) at 80 °C in 3 static cycles that were 5 min each, with purge time settings of 30 s and a flush volume of 30%. The obtained extract was evaporated to dryness under a vacuum and was further used for the isolation of MGN on a centrifugal partition chromatograph (CPC) (SCPC-250, Armen Instruments, Saint Ave, France). The fractionation of the dried extract was performed using the biphasic solvent system composed of chloroform:methanol:water (4:3:3 *v*/*v*/*v*), with the addition of 20 mM of triethylamine and hydrochloric acid to the organic and aqueous phase, respectively, using elution–extrusion fractionation in the descending mode. The fractionation was performed at a rotation speed of 1300 rpm with a flow rate of 6 mL/min for the first 50 min (elution mode) and at a flow rate of 8 mL/min for the following 36 min (extrusion mode), whereas MGN was eluted from the column at 63 min. All fractions were analyzed for the presence of MGN using the HPLC-ESI-QTOF-MS/MS instrument (composed of the HPLC chromatograph of 1200 Series and the ESI-QTOF-MS/MS detector of G6530B series, Agilent Technologies, Santa Clara, CA, USA). Those with high MGN content were joined, evaporated to dryness and used for the bioactivity assay. The mass chromatograms showing the purity of the isolate, together with its fragmentation pattern and CPC chromatogram, are presented in the Supplementary File (Figures S1–S3) and were obtained under the previously reported conditions [12].

### 4.2. Cell Lines Culture

The human GC cell lines 23132/87 (ACC-201) and MKN-74 were purchased from the Leibniz Institute DSMZ (German Collection of Microorganisms and Cell Cultures, Brunswick, Germany) and AcceGen Biotechnology (Fairfield, NJ, USA), respectively. The human GC cell lines AGS (CRL-1739) and NCI-N87 (CRL-5822) were obtained from the American Type Culture Collection (ATCC). AGS cells were incubated in F-12K Medium (Kaighn’s Modification of Ham’s F-12 Medium) (ATCC) containing 2 mM L-glutamine, 1500 mg/L sodium bicarbonate and supplemented with 10% fetal bovine serum (Sigma-Aldrich, St. Louis, MO, USA) and antibiotics such as penicillin (100 IU/mL) and streptomycin (100 μg/mL) (Sigma-Aldrich). The remaining cancer cell lines were grown in RPMI 1640 medium supplemented with 10% fetal bovine serum (Sigma-Aldrich) and antibiotics (100 IU/mL of penicillin and 100 μg/mL of streptomycin) (Sigma-Aldrich). Cancer cell lines were cultured in a humidified 5% CO_2_ atmosphere at 37 °C.

### 4.3. MTT Assay

The human GC cells ACC-201, AGS, MKN-74 and NCI-N87 were placed in 96-well plates and allowed to adhere overnight. On the following day, the growth medium was removed, and the cells were treated with a series of tested MGN and DCT dilutions (2.0–75 and 0.0004–0.0404 µg/mL, respectively) for 72 h at 37 °C. The highest concentration of dimethyl sulfoxide (DMSO) used as the vehicle control does not exceed 0.1%, and it does not influence the viability of cancer cells. Subsequently, a solution of MTT (5 mg/mL) was added to each well, and the cells were further incubated for 3 h at 37 °C. Tetrazolium salt was reduced to violet formazan derivatives via the succinate dehydrogenase of metabolically active cells. Insoluble formazan compounds formed inside the cells were extracted with a sodium dodecyl sulfate buffer (10% SDS in 0.01 N HCl) overnight to achieve a quantitative assessment of the amount produced. The absorbance of the samples was measured at 570 nm using Infinite M200 Pro microplate reader (Tecan, Männedorf, Switzerland). Cell viability was presented as a percentage relative to the untreated control cells.

### 4.4. BrdU Incorporation Assay

The human GC cells ACC-201, AGS, MKN-74 and NCI-N87 were placed in 96-well plates and allowed to adhere overnight. On the following day, the growth medium was replaced with fresh medium, and the cells were exposed to a series of tested MGN dilutions (2.0–75 µg/mL) for 72 h. Cell proliferation was quantified according to the manufacturer’s instruction, Cell Proliferation ELISA, BrdU (colorimetric) (Biorbyt, Cambridge, UK). Briefly, BrdU reagent was added to the medium, followed by Fixative/Denaturing Solution for 30 min at room temperature. Incorporated BrdU in the place of thymidine in the cell’s DNA under division was detected using mouse monoclonal anti-BrdU for 1 h at room temperature, followed by horseradish peroxidase (HRP)-linked goat anti-mouse IgG secondary antibody and tetramethylbenzidine (a HRP substrate) for 30 min at room temperature. The absorbance was measured at 450 nm using Infinite M200 Pro microplate reader (Tecan, Männedorf, Switzerland). Results are presented as a percentage of BrdU incorporation vs. control cells (indicated as 100%).

### 4.5. Cell Cycle Analysis

Human GC cells ACC-201, AGS, MKN-74 and NCI-N87 were seeded in 6-well plates and allowed to adhere overnight. On the following day, the growth medium was removed, and the cells were treated with indicated concentrations of MGN (10–40 µg/mL) for 72 h. After that, the cells were washed with phosphate-buffered saline (PBS) and fixed in 70% ethanol at 4 °C overnight. Then, the cells were washed with PBS and stained with propidium iodide, utilizing PI/RNase Staining Buffer (BD Biosciences, Heidelberg, Germany) for 15 min in the dark. Cell distribution in different stages of the cell cycle was performed through the use of the FACS CaliburTM flow cytometer (BD Biosciences, Mountain View, CA, USA).

### 4.6. Active Caspase-3 Analysis

Human GC cells ACC-201, AGS, MKN-74 and NCI-N87 were seeded in 6-well plates and allowed to adhere overnight. On the following day, the growth medium was replaced with fresh medium, and the cells were exposed to indicated concentrations of MGN (10–40 µg/mL) for 72 h. Apoptotic cells were analyzed using PE Active Caspase-3 Apoptosis Kit (BD Biosciences) according to the manufacturer’s instructions. Briefly, the cells were collected, washed with cold PBS and then incubated in Cytofix/Cytoperm Solution for 20 min on ice. After two washes, the cells were incubated with Active Caspase-3 Antibody in Perm/Wash Buffer for 30 min at room temperature. Finally, the cells were resuspended in Perm/Wash Buffer and quantitative analysis of 10,000 cells per sample was performed through the use of FACS CaliburTM flow cytometer (BD Biosciences, Mountain View, CA, USA).

### 4.7. Isobolographic Analysis

Interactions between MGN and DCT analyzed after 72 h in four different GC cell lines (ACC-201, AGS, MKN-74 and NCI-N87) were characterized by means of an isobolographic analysis in the MTT assay. The percentage inhibition of cell viability accompanied by the specific concentrations of the tested substances (MGN and DCT) were transferred to the MS Excel spreadsheet, and the data were computed using the log–probit method [52]. Next, the concentration–response effect graphs for the tested drugs (MGN and DCT) were drawn, and the IC_50_ values for every tested drug were calculated. The cooperative effect of two concentration–response lines was tested for the MGN and DCT combination, as previously described [53,54]. The calculations of IC_50_ values (±SEM) were computed with formulas originally derived from the log–probit method [52] and adapted to the in vitro conditions, as described earlier [53,54]. The concentration–response lines for MGN and DCT were not parallel, and thus, the isobolographic additivity was illustrated graphically as an area bounded by two (upper and lower) lines of additivity [53,54]. From the experimentally denoted IC_50_ values for MGN and DCT in the MTT assay, median additive inhibitory concentrations (IC_50add_) for the mixture (at the fixed ratio of 1:1 of MGN and DCT) in various cell lines (ACC-201, AGS, MKN-74 and NCI-N87) were calculated, as described previously [55,56]. More information on isobolographic analysis can be found in the literature [56,57,58].

### 4.8. Zebrafish Studies

For all in vivo studies, we followed the European Community Council Directive of November 2010 for Care and Use of Laboratory Animals (Directive 2010/63/EU) and the National Institute of Health Guidelines for the Care and Use of Laboratory Animals guidelines. Only zebrafish larvae up to 96 hpf were used. Ethical permission to perform experiments on zebrafish embryos and larvae up to 120 hpf is not required, but we made all efforts to minimize the number of fish used and their suffering. Right after the experiments, larvae were euthanized using a solution of 15 µM tricaine.

For in vivo analysis, zebrafish embryos of the AB strain were purchased from the Experimental Medicine Centre (Medical University of Lublin, Lublin, Poland). They were housed under standard conditions (28.5 °C, 14 h light/10 h dark cycle) in an incubator.

To assess the impact of MGN on developing organisms, we conducted a zebrafish embryo toxicity test in accordance with the guidelines of the Organization for Economic Cooperation and Development (OECD) (test no. 236) [59] with minor modifications, as described previously [60]. At first, zebrafish embryos were screened and sorted one hour after fertilization. Only perfectly transparent and fertilized embryos were transferred to plastic well plates. In a single well, at least 3 embryos were maintained in a volume of 400 µL of medium with the addition of MGN (0, 100, 175, 250, 500 or 750 µg/mL). Developing embryos were incubated in the MGN solutions until 96 hpf. The amounts of MGN absorbed by zebrafish were measured in 96 hpf-old larvae (n = 50 larvae/sample, three samples/dose) through the use of HPLC-MS. The larvae were first washed in medium prior to homogenization with the additive of acetonitrile:water mixture in equal proportions (50:50 *v*/*v*). The homogenate was centrifuged at 20,000 rpm for 20 min, and the supernatant was pipetted, filtered through a nylon syringe filter (nominal pore size of 0.1 µm diameter) and subjected to HPLC-MS analysis in the positive ionization mode [60,61].

The following phenotypic features were scored in larvae exposed to different doses of MGN: (I) hatchability at 72 and 96 hpf, (II) morphological anomalies at 96 hpf, and (III) escape response at 96 hpf. Morphological analysis included the presence/lack of swim bladder, body axis/shape, presence/lack of pericardial edema, jaw development, normal/necrotic yolk sac, presence/absence of hemorrhage and changes in pigmentation [60]. For photographs, each larva was anaesthetized and mounted on a glass slide. To evaluate the muscle performance and function in MGN-incubated larvae, in a separate cohort of 96 hours-old larvae, we assessed escape response by touching the tail with the tip of a needle [62].

Locomotor activity was examined to determine whether chronic MGN exposure may induce neurotoxic effects in fish. Noldusapparatus (EthoVision XT, Wageningen, The Netherlands) was used for this purpose. After 95 h of incubation in different doses of MGN (100, 175 or 250 µg/mL), each larva was placed individually in the wells of a 48-well plate filled with 300 µL of the medium. After 15 min of habituation, larval activity was tracked in the light phase for a period of 30 min. The distance covered by each larva in millimeters (mm) was assessed.

### 4.9. Statistical Analysis

For the in vitro experiments, statistical analyses were performed using a one-way ANOVA test followed by Tukey’s post hoc test using GraphPad Prism (version 8.0, San Diego, CA, USA). The unpaired Student’s *t*-test with Welch’s correction was used to statistically compare the experimentally derived IC_50mix_ values (for the mixture of MGN and DCT) with their respective, theoretically calculated and presumed additive IC_50add_ values, as recommended elsewhere [55,63], using GraphPad Prism (version 8.0, San Diego, CA, USA). All the isobolograms were drawn in the MS Excel spreadsheet. In the zebrafish assays, all studies were performed 3 times, and the data were pooled together. For statistical analysis, we used a Chi-squared test, Fisher’s test or one-way ANOVA with Tukey’s post hoc test. The data were analyzed and figures were generated with the aid of GraphPad Prism 9.3.1 version.

## 5. Conclusions

Collectively, the presented results demonstrate that MGN inhibited the proliferation of ACC-201 and AGS derived from primary tumor and metastatic MKN-74 and NCI-N87 human GC cell lines through cell cycle arrest and apoptosis induction without any undesirable changes in the development of larval zebrafish at the tested concentrations. Taken together, these findings provide the foundation for the possibility of using MGN as a potential therapeutic approach for GC and merits further investigation, which may pave the way toward clinical uses of MGN.

## Figures and Tables

**Table 1:** The anti-proliferative effects of MGN and DCT in four various GC cell lines detected in vitro via the MTT assay after 72 h incubation. The IC_50_ values (μg/mL) are presented as means ± SEM.

Drug/Cell Line	ACC-201	AGS	MKN-74	NCI-N87
MGN	15.75 ± 2.79	17.19 ± 3.59	34.82 ± 6.04	33.31 ± 5.00
DCT	0.0028 ± 0.0009	0.0026 ± 0.0012	0.0049 ± 0.0021	0.0090 ± 0.0029

**Table 2:** Quantitative analysis of GC cell lines positive for cleaved caspase-3 after treatment with MGN. Statistical differences were analyzed using Student’s *t*-test (* *p* < 0.05; *** *p* < 0.001; **** *p* < 0.0001 vs. control).

Cell Line	Control	10 µg/mL	20 µg/mL	30 µg/mL	40 µg/mL
ACC-201	3.623 ± 0.416	6.770 ± 0.866	13.150 **** ± 0.962	16.830 **** ± 1.258	23.630 **** ± 1.758
AGS	1.222 ± 0.336	2.390 ± 0.411	4.172 ± 0.422	12.070 **** ± 1.451	24.980 **** ± 2.048
MKN-74	1.580 ± 0.065	2.633 ± 0.069	4.148 *** ± 0.316	5.636 **** ± 0.398	7.481 **** ± 0.725
NCI-N87	1.396 ± 0.086	2.948 * ± 0.133	5.233 **** ± 0.310	8.568 **** ± 0.652	10.030 **** ± 0.398

**Table 3:** Cell cycle analysis of GC cell lines treatment with MGN. Statistical differences were analyzed using Student’s *t*-test (* *p* < 0.05; ** *p* < 0.01; *** *p* < 0.001; **** *p* < 0.0001 vs. control).

Cell Line	Phase	Control	10 µg/mL	20 µg/mL	30 µg/mL	40 µg/mL
ACC-201	subG1	9.91% ± 2.376	18.52% * ± 1.414	26.06% *** ± 1.399	38.50% **** ± 1.886	51.43% **** ± 2.072
	G1	67.05% ± 1.547	62.76% *** ± 2.434	57.23% **** ± 2.112	49.55% **** ± 1.820	39.27% **** ± 3.408
	S	9.68% ± 1.596	8.74% ± 3.423	8.33% ± 1.647	6.16% **** ± 1.340	5.61% **** ± 1.874
	G2	13.12% ± 5.442	9.96% **** ± 3.707	8.34% **** ± 4.237	5.87% **** ± 5.399	3.74% **** ± 1.615
AGS	subG1	0.55% ± 2.249	1.19% ± 2.437	2.17% ± 2.519	2.17% ± 3.000	2.98% ± 2.448
	G1	61.82% ± 4.089	58.09% ± 8.195	53.06% ± 12.980	43.08% * ± 14.330	38.15% ** ± 13.350
	S	16.32% ± 3.114	17.62% ± 3.599	21.07% * ± 2.593	21.55% * ± 2.265	19.89% ± 2.653
	G2	21.50% ± 7.406	23.35% ± 12.110	24.06% ± 16.000	31.77% * ± 17.630	39.34% ** ± 15.720
MKN-74	subG1	0.64% ± 2.185	1.60% * ± 2.688	2.28% *** ± 2.818	3.59% **** ± 2.787	5.60% **** ± 1.002
	G1	61.92% ± 1.587	69.07% *** ± 1.886	73.81% **** ± 4.227	65.90% ± 3.533	50.20% **** ± 5.303
	S	18.08% ± 1.710	13.36% **** ± 0.622	9.97% **** ± 1.867	7.72% **** ± 1.822	8.25% **** ± 2.280
	G2	19.66% ± 2.576	16.23% ± 1.928	14.14% ** ± 2.076	22.92% ± 2.455	35.94% **** ± 6.691
NCI-N87	subG1	1.88% ± 2.316	2.41% ± 2.189	3.93% **** ± 2.591	6.33% **** ± 2.772	9.47% **** ± 1.163
	G1	68.25% ± 5.515	69.39% ± 3.658	70.17% ± 2.905	67.45% ± 3.535	64.37% ** ± 3.651
	S	12.69% ± 4.189	11.81% ± 3.808	10.44% ** ± 6.685	10.12% *** ± 7.739	9.24% **** ± 5.603
	G2	17.28% ± 2.893	16.52% ± 2.770	15.56% * ± 2.419	16.21% ± 1.874	17.06% ± 2.426

**Table 4:** Isobolographic analysis of interactions between MGN and DCT (at the fixed ratio of 1:1) for nonparallel concentration–response effects in various GC cell lines.

Cell Line	IC_50exp_ (n_exp_) (µg/mL)	Lower IC_50add_ (n_add_) (µg/mL)	Upper IC_50add_ (n_add_) (µg/mL)	*t*-Test
ACC-201	5.70 ± 1.04 (72)	6.60 ± 1.16 (176)	9.70 ± 1.21 (176)	t = 0.578; df = 219.6 *p* = 0.564
AGS	6.86 ± 1.22 (72)	6.63 ± 1.61 (176)	10.56 ± 1.70 (176)	t = 0.114; df = 239.2 *p* = 0.909
MKN-74	11.43 ± 2.34 (90)	11.38 ± 4.03 (176)	23.43 ± 4.11 (176)	t = 0.011; df = 255.7 *p* = 0.991
NCI-N87	7.66 ± 2.19 (90)	8.82 ± 3.41 (176)	24.56 ± 3.82 (176)	t = 0.286; df = 261.6 *p* = 0.775

The IC_50_ values (in µg/mL ± SEM) for the mixture of MGN with DCT were determined experimentally (IC_50exp_) in the in vitro MTT assay. The IC_50add_ values were calculated from the lower and upper isoboles of additivity; n_mix_: total number of items experimentally determined; n_add_: total number of items calculated for the additive two-drug mixture.

**Table 5:** Hatching rate (%) of larvae chronically exposed to different concentrations of MGN, scored at 72 and 96 hpf. Data pooled from 3 independent experiments. hpf = hours post-fertilization.

Concentration (µg/mL) (n per Group)	0 (n = 35)	100 (n = 32)	175 (n = 30)	250 (n = 36)	500 (n = 30)
72 hpf	97.14%	100%	100%	52.77%	23.33%
96 hpf	100%	100%	100%	94.44%	86.66%

## Data Availability

Data are contained within the article.

## Supplementary Materials

The supporting information can be downloaded at: https://www.mdpi.com/article/10.3390/ijms242115511/s1.
