# Supplementary material for: Heterogeneous Cellular Response of Primary and Metastatic Human Gastric Adenocarcinoma Cell Lines to Magnoflorine and Its Additive Interaction with Docetaxel
Source: Int J Mol Sci. 2023 Oct 24;24(21):15511. doi: 10.3390/ijms242115511 (PMC10647589; doi:10.3390/ijms242115511)

SUPPLEMENTARY FILE

Figure S1. The chromatogram of magnoflorine isolated from *Berberis vulgaris* root

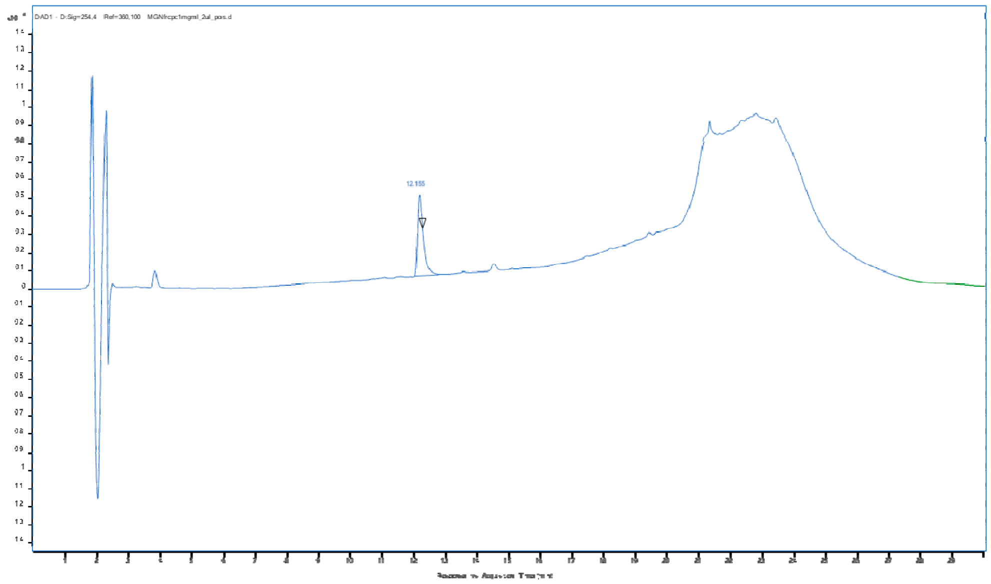

Figure S2. The MS/MS chromatogram showing the fragmentation of the isolated magnoflorine

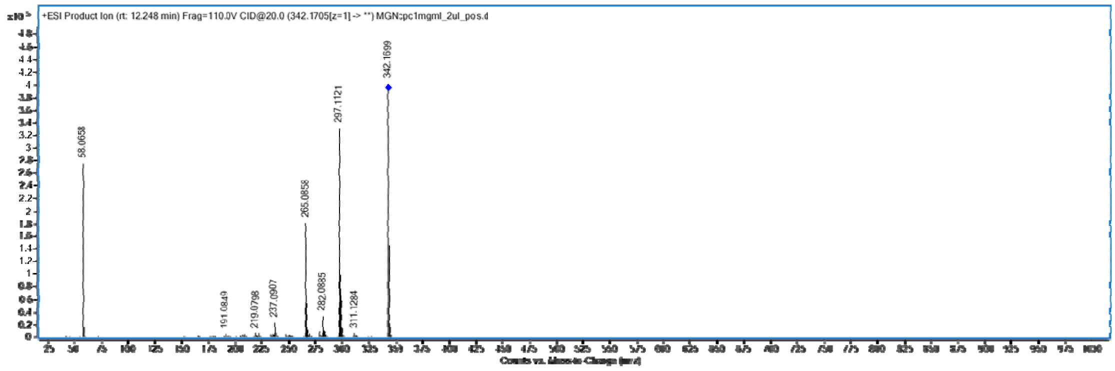

Figure S3. Chromatogram registered by the CPC instrument with the peak of magnoflorine eluted after 63 min

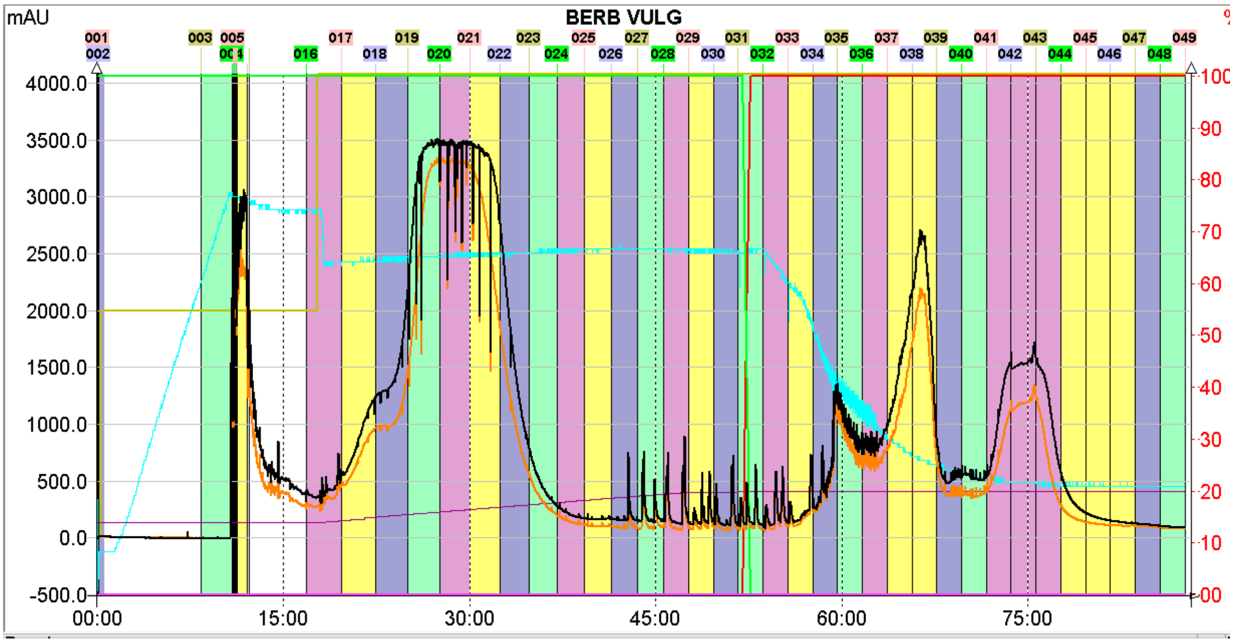

Supplement: Supplementary file 1 [file ijms-24-15511-s001.zip › ijms-2550231-supplementary.pdf]
